# Sucrose Esters as Oleogelators in Mono or Binary Structured Oleogels Using Different Oleogelation Routes

**DOI:** 10.3390/gels9050399

**Published:** 2023-05-10

**Authors:** Thais Lomonaco Teodoro da Silva, Sabine Danthine

**Affiliations:** Science des Aliments et Formulation, Gembloux Agro-Bio Tech, ULiège, 5030 Gembloux, Belgium

**Keywords:** sucrose ester, oleogels, binary structuration, direct dispersion, foam-template, solvent exchange

## Abstract

Sucrose esters (SE) have been investigated as structuring agents in oleogels. Due to the low structuration power of SE as single agent, this component has recently been explored in combination with other oleogelators to form multicomponent systems. This study aimed to evaluate binary blends of SEs with different hydrophilic-lipophilic balances (HLBs) with lecithin (LE), monoglycerides (MGs) and hard-fat (HF), according to their physical properties. The following SEs, SP10-HLB2, SP30-HLB6, SP50-HLB11, and SP70-HLB15, were structured using three different routes: “traditional”, “ethanol” and “foam-template”. All binary blends were made using a 10% oleogelator in 1:1 proportion for binary mixtures; they were then evaluated for their microstructure, melting behavior, mechanical properties, polymorphism and oil-binding capacity. SP10 and SP30 did not form well-structure and self-standing oleogels in any combination. Although SP50 showed some potential blends with HF and MG, their combination with SP70 led to even more well-structured oleogels, with a higher hardness (~0.8 N) and viscoelasticity (160 kPa), and 100% oil-binding capacity. This positive result might be attributed to the reinforcement of the H-bond between the foam and the oil by MG and HF.

## 1. Introduction

Oleogels are a mixture of oleogelators and vegetable oils, where the liquid oil is entrapped within the three-dimensional network of oleogelators, acquiring a semi-solid texture (gel) [1]. Oleogels can be structured using different structuration routes depending on their solubility strategies, categorized as direct or indirect dispersion [2]. The direct dispersion approach is the simple dissolution of the fully melted oleogelators into the oil. After complete dispersion, the mixture of oil and oleogelator is cooled down, forming a solid structure network that entraps the liquid oil [3,4]. Indirect dispersion is especially needed for hydrophilic polymers, which cannot be directly dispersed in oil, where another solvent is used to dissolute the gelator, mostly water. After stripping off the water from hydrated polymer solutions, a direct dispersion or oleogel is formed [2,5]. For instance, the following methods fall into this category: the emulsion-templated method [5], foam-templated method [6], and solvent exchange [7]. The emulsion-template approach involves a first emulsion preparation with further evaporation of the water phase [5]. The foam-template approach requires the dissolution and homogenization of the gelator in water; this is then freeze-dried and the remaining gelator is further incorporated in liquid oil [6]. For the solvent exchange, instead of using water, the primarily dissolution is carried out in an organic solvent, such as ethanol or hexane. The solvent is then evaporated and the gelator is further used to structure oils [7]. The first dissolution step of the hydrophilic gelators in all those methods improves their solubility in oil and makes it possible to form oleogels [8].

The physical properties of each gel, such as their viscosity, hardness, and melting point, are strongly related to the oleogelator that is used [9]. Those properties are also correlated with the future application of the oleogel, and different oleogelators and interactions have been found to tailor these properties [10]. The main evaluated oleogelators reveal different waxes [11], monoglycerides (MG) [12], lecithin (LE) [4], sorbitan esters [13], sucrose esters (SEs) [14], phytosterols [15], ethylcellulose [16], and high-melting point triacylglycerols [17], among others. Oleogels can be applied in different types of food products, such as margarine [18], chocolate [19], chocolate spreads [20], ice cream [21], meat products [22], cakes [23], fillings [24], peanut butter [25], and dairy products [26,27].

SEs are fatty acids esterified with sucrose; they can form structures ranging from monoesters to octa esters. The properties of the SE, such as its hydrophilic–lipophilic balance (HLB), emulsification capacity and stability, and solubility, are influenced by the type of fatty acid (C8-C22), saturation and length, as well as by the degree of substitution (mono, di, tri-esters). The current use of SEs in food products is as emulsifiers and stabilizers. However, they can also improve aeration, tailor the crystallization of sugar and fats, and increase lubrification. [28]. Few studies have explored the potentialities of SEs in oleogels [14,29,30,31]. This can be attributed to the low structuration power of SEs to form oleogels at concentrations below 25% [31] and their solubility in the lipid phase, which seems to be improved by the use of alternative routes to structure the oleogel [14], or by combining the SE with others oleogelators, such as LEs [30] or ascorbyl palmitate [31].

MGs are lipid molecules with an amphiphilic character formed by one fatty acid esterified on the glycerol. Different MGs vary in their type and the length of the carbon chain of fatty acids [32]. As well as structuring emulsions, they can also self-assemble upon cooling and form oleogel [33]. MGs are one of the most evaluated oleogelators; they have been successfully explored as solo oleogelators, forming stable oleogels with good physical properties [12,34,35,36] or in diverse multicomponent systems [37,38,39,40].

Les also have an amphiphilic character, and is composed of different fractions and portions of phosphatides. LEs can be obtained from animal (eggs, milk) or vegetable (soybean, sunflower, etc.) sources [41]. Although LEs can serve as oleogelators in non-organic phase, they are not powerful oleogelators when used alone, requiring a higher concentration of the oleogelator (>20%) [42]. Nevertheless, some synergic combinations of LEs and other components have been found [4,30,42,43].

Hard fats (HFs) are liquid oils that have been transformed into fully saturated fats by full hydrogenation. They often present a homogeneous triacylglycerol composition, with a high melting point, and can be used as low-cost industrial additives [44]. HFs have been used to structure oleogels in diverse hybrid systems [13,17,45,46].

Previously, our group has demonstrated that SEs can form oleogels using different structuration routes, based on the HLB value [14]. Nonetheless, the oleogels formed, even using the optimized route, were still very soft. Therefore, the objective of this work was to evaluate the potential of the same SEs, using their optimized routes, in binary structured oleogels combined with MG, LE, or HF.

## 2. Results and Discussion

### 2.1. Microscopy and Visual Observation

**Figure 1:**
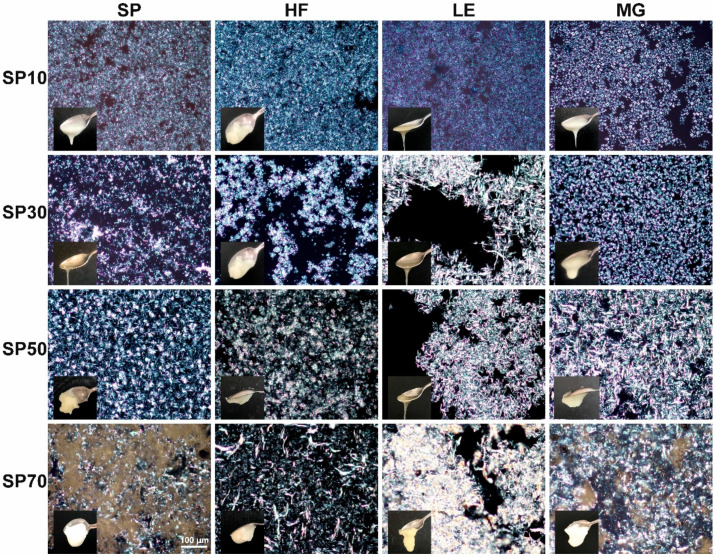
Macro- and microstructure of the SEs (SP10, SP30, SP50 and SP70) and their blends with hard fat (HF), lecithin (LE) and monoglycerides (MGs), 20× magnification.

In Figure 1, the macroscopic and microscopic structure of the SEs and their blends can be seen. Even after optimizing the routes [14], the different SEs do not form self-sustainable oleogels if using 10% max. This is in agreement with a previous report where at least 20% of SEs with a different HLB (1 and 9) were required to form stable gels [29]. The binary mixture seems to improve the visual structuration in some cases, depending on the system.

SP10 showed very small polydisperse globular crystals in a compact and dense structure, which can be seen in Figure 1 and Figure S1 (Supplementary Material). In blends, HF and LE seem to have the same microstructure as SP10 alone. However, MG changed the SP10 network, forming a crystalline structure with a Maltese cross (Figure S1), which was previously observed for other SEs with a very low HLB [47]. Even though SP10 forms many small crystals, they are globular, and this type of crystal is not correlated with the formation of a well-connected crystal network [30]. For this reason, they do not form well-entrapped and self-sustainable oleogels. Although HF seems to improve this behavior; nonetheless, LE and MG still form very liquid gels. In a previous study, an SE (HLB 2) was mixed with sunflower LE, and at ratios of 8:2 and 6:4, the blend SE:LE maintained solid-like properties at room temperature. Nevertheless, at ratios of 5:5 and 3:7, samples became liquid when remaining at room temperature for 10 min; moreover, LE or SE alone could form gels [29]. SE (HLB 2) was blended with ascorbyl palmitate, and 12% of oleogelators in a 1:1 blend ratio were required to form visually structured and stable oleogels. Lower concentrations (8 and 10%), and different ratios (3:1 and 1:3) were also tested [31]. This led us to hypothesize that a higher proportion of oleogelators would be needed to form self-sustainable gels for SP10 at room temperature, or a specific untested ratio.

SP30 alone showed a similar structure to SP10, but the polydisperse crystals were bigger and occurred in lower amounts. Compared to the SP10, they also showed some agglomerations, with spots where a higher density of crystals was observed. The HLB of emulsifiers directly affects the crystallization rate and type of crystalline structure formed in the oleogel. A lower HLB, as seen in the SP10, is likely a contributing factor to the growth of numerous small crystals [31]. SP30 blends have diverse microstructures. The HF blend showed some crystals clusters composed of globular crystals and some Maltese crosses. LE blends showed big needles, and MG showed very tiny needles and some spherulites. The addition of MG to beeswax oleogels also revealed a combination of spherulitic crystals with tiny needle-like crystals in the background; when this crystal combination was present, the sample did not show very good structuration [37]. A similar microstructure was observed for SP30MG blends, as shown in Figure 1.

The SP50 ethanol network was formed by two types of crystals with needles and a globular morphology. Although formed by two types of crystals, they were well-connected. In oleogel blends of SP50 with HF, only smaller-needle crystals were observed, and these needles were mostly agglomerated in clusters. In blends with LE or MG, only needles were observed; however, much larger needles were observed. In the LE blend, few needles were observed, with lots of empty space (no crystals); nonetheless, in the MG blend, the needles were well-connected and distributed. Regarding the visual observation, SP50 showed two self-sustainable oleogels when blended with HF or with MG. Surprisingly, the blend with LE negatively affected the structuration power of the SE. These results confirm the needles’ potential to structure oleogels [48]. Blends of MG with phytosterols also increased the size and amount of needles, forming the most common agglomerate version of the needles, the spherulites. [49]. The higher-density network that was formed was responsible for entrapping and limiting the oil-liquid diffusion out of the crystalline network [40]. Observing our results, it seems that the size of the needles is less important than how they are distributed in the crystalline network for self-sustainable gels, since MG and HF showed very different needle sizes. However, the proximity of those needles and the absence of empty spaces in the oleogel network play an important role, as observed by the lack of structure in SP50-LE.

The SP70 single-structured oleogel presented a combination of crystalline and non-crystalline networks. In the blends, this non-crystalline phase was only observed in small amounts in the blend with MG. MG was previously used to strengthen the oleogelation of the cellulose ester foam-template oleogel, which formed granular crystalline particles around the non-crystalline network of the cellulose ester, forming a double-structure oleogel network [50]. Other LE and HF blends did not shown this structure. Moreover, the needle backgrounds were observed in all cases. For HF, both big (as for SP70 alone) and smaller needles were observed, suggesting that the HF is connecting the large SP70 needles. Only big needles were observed for the LE blend; however, they were distributed differently forming a bigger cluster of agglomerated big crystals. For the MG blend, only smaller needles were observed. Regarding visual structure, all SP70 blends showed an improvement in the visual structuration, with a detachment from the SP70-HF blend. The SP70-LE blend showed an improved structure, although it was still soft. This is in agreement with previous research on phytosterols and LE blends, which conclude that there is no solid behavior in LE blends below 10% of the total oleogelator [51].

### 2.2. Oil Loss

**Figure 2:**
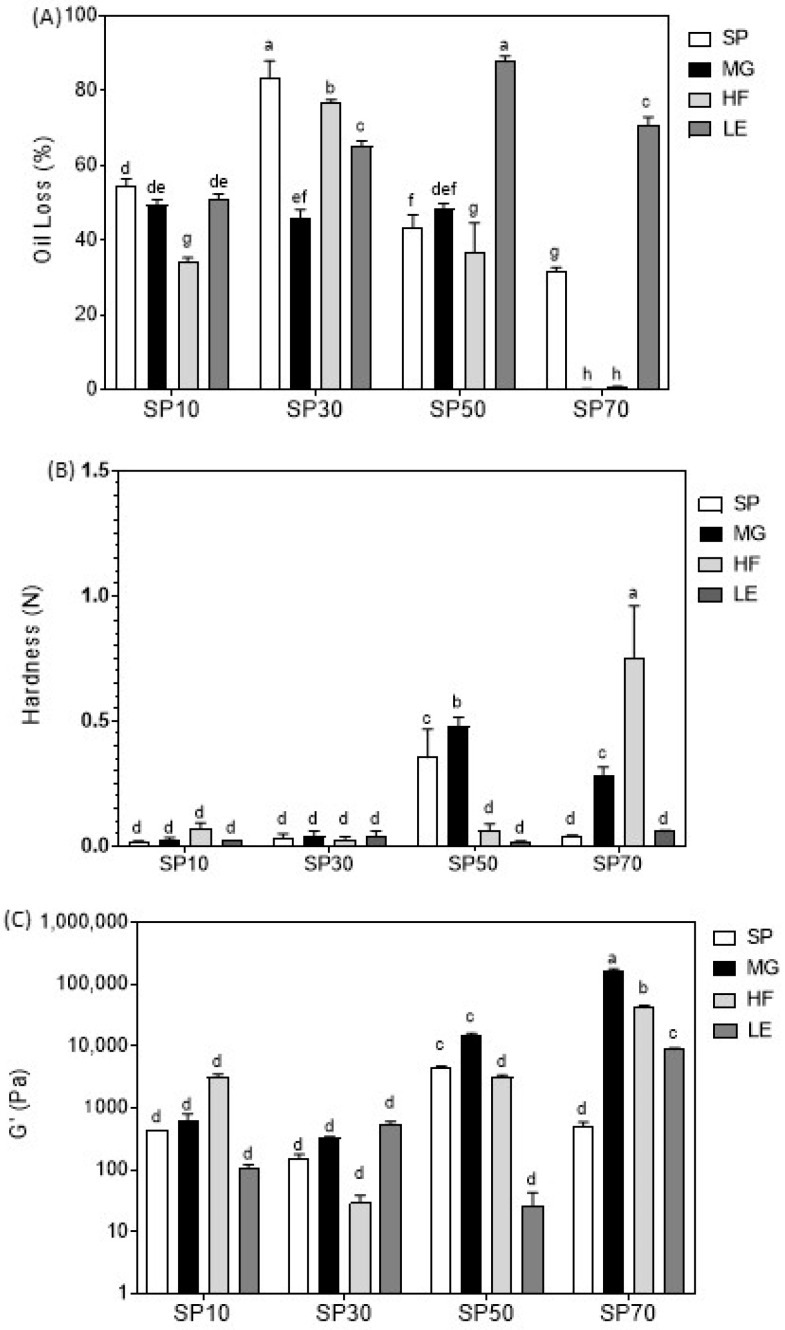
Oil loss (**A**), hardness (**B**), viscoelasticity (**C**), of the sucrose esters oleogels (SP10, SP30, SP50 and SP70) and their blends with monoglycerides (MG), hard fat (HF) and lecithin (LE). Samples followed by the same letter in the same column are not statistically different from each other based on Tukey test (α = 0.05).

Oil loss results can be seen in Figure 2A. SEs presented a high level of oil loss, regardless of the type of SE or the oleogelation route used. According to the literature, the values can be as high as 80% (SP30), or lower (~30%, SP70) if the oleogelation route is well-designed for the considered type of SE [14].

It is clear that the investigated blends behave differently depending on the SE that is used. SP10 only showed an improvement in oil loss when blended with HF. The MG and LE blends showed a similar oil-binding capacity as SP10 alone (*p* > 0.05). SP30 showed a significant improvement in oil loss in all blends (*p* < 0.05). The lower oil loss was observed for SP30MG. SP50 blends showed an improvement when blended with HF, no significant difference with MG, and, surprisingly, an increase in oil loss was observed in the LE blend.

The most promising blends concerning oil loss results were the SP70 blends: mixing SP70 with HF or MG oleogelators led to an oil loss of lower than 0.5%. However, LE does not seem to have the same effect on the foam-structured SP70 oleogel. This leads us to conclude that LE is not a good candidate for structuring oleogels with the investigated SEs, since all SEs, when mixed with LE, did not show a good oil-binding capacity. LE blends (SP10 excepted) showed a poorly connected network, full of crystalline agglomerates with large intermolecular empty spaces (Figure 1), which explained the very low oil-binding capacity of the LE blends. This is in agreement with Blake et al. [52], who reported that, when the crystals were covering all spaces and consistently dispersed, the volume of dripping unbound oil reduced.

However, for SP10 and SP50, HF was the best component to add and, together with MG, this was also true for SP70. This could be correlated with the microstructure results, since all blends containing HF showed a well-connected and organized crystalline network, except for SP30HF (Figure 1). MG showed a medium compatibility with SE to form oleogels. This can be explained by the lack of structure of the SE itself. More structured SE oleogels were obtained by the addition of MG; this was expected, since MG alone already shows a good oil-binding capacity [53].

### 2.3. Hardness and Viscoelasticity

The hardness of the oleogels is shown in Figure 2B. Although the addition of MG, HF, and LE induced some changes in microstructure and oil loss for SEs SP10 and SP30, their hardness was very low, and no difference was found, regardless of the type of oleogelator used in the blend.

Samples SP50HF, SP50LE, SP70, and SP70LE showed a similar, very low hardness to the SP10 and SP30 oleogels. A higher hardness was found for samples SP50, SP50MG, SP70MG, and SP70HF, with an emphasis on sample SP70HF., suggesting a synergic interaction between the high hydrophilic SEs with HF and MG.

Figure 2C shows the viscoelasticity of the oleogels. Similar to the results obtained for hardness, samples SP50, SP50MG, SP70MG, and SP70HF showed a higher elastic modulus (G′). The difference in this parameter is that a higher G′ was found for blend SP70MG, not SP70HF, and that SP70LE showed a significant improvement in G′ compared to the low-viscoelasticity samples (all samples followed by letter d). This is in accordance with the study of Jiang et al. [50], where MG crystal networks significantly contributed to the improvement in the rheological properties of foam-template oleogels, which we attributed to the reinforcement of the H-bonds between the SP70 foam by the high-melting-point MG and HF.

A considerable increase in viscosity when SE was added to concentrations of 10–15% was reported by Nelen et al. [28]. However, using 10% SE or in binary blends, SP10 and SP30, using direct dispersion, did not improve considerably hardness, suggesting that either the amount of SE was not enough or that SE under direct dispersion does not form hard gels in concentrations below 10%, or when combined with MG, HF, and LE.

### 2.4. Melting Properties

**Figure 3:**
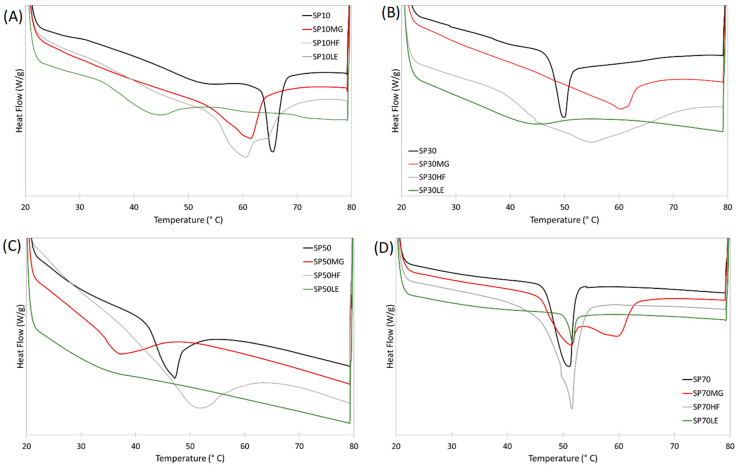
Melting curves of the sucrose esters oleogels (**A**) SP10, (**B**) SP30, (**C**) SP50 and (**D**) SP70 and their blends with monoglycerides (MG), hard-fat (HF) and lecithin (LE).

The melting curves of the oleogels are presented in Figure 3. We recently reported that oleogels made of MG and HF as a single oleogelator, in 10% proportion, showed a T_p_ of 64.7 °C and 55.5 °C, and enthalpy of 18.1 and 15.6 J/g, respectively. On the other hand, LE alone showed no peak between 20 and 80 °C during melting [38]. SP10 and their blends are shown in Figure 3A and Table 1; the SP10 oleogel showed a higher T_p_. All SP10 blends showed a lower T_p_ compared to SP10 alone; however, compared to HF and LE alone, the T_p_ of the blends was higher. Blends with MG and HF showed a similar T_p_; conversely, the HF blend showed a shoulder at a higher temperature (65.1 °C), suggesting that SP10 and HF do not co-crystallize. A previous evaluation of an SE:LE system showed a reduction in T_p_ and enthalpy when they were blended [30]; nonetheless, the LE used in this case showed no peak under the evaluated conditions, which makes it harder to assume this. However, based on the microstructure, oil loss, and mechanical properties results just discussed, LE might be diluting SE crystals instead of self-assembling with them (see the lack of structure in Figure 1 and very low enthalpy in Table 1).

SP30 blends’ melting curves can be seen in Figure 3B and Table 1. SP30 oleogel showed a lower T_p_ than SP10. As the melting curves of MG and HF were very close to their T_p_ when used as a single oleogelator, we can conclude that SP30 and MG/HF are probably not bounding. LE, in this case, does not significantly reduce the enthalpy but still does not assemble with the SE.

The SP50 oleogel showed a similar T_p_ to SP30 (*p* < 0.05). SP50MG showed a significantly lower T_p_ than both SP50 (51.0 °C) and MG (64.7 °C) single-component oleogels. According to previous multi-component oleogels studies, this occurs due to the co-crystallization [17] or self-assembly [32] of the oleogelators in the oleogel and is followed by an increase in the mechanical properties and more organized crystalline network. The blend with HF showed a proportional T_p_ between HF and SP50, and LE showed a very small peak.

The group of SP70 based oleogels was the only one where all the blends showed the same T_p_ as the SP70 mono-component oleogel. This suggests that SP70 is directing the oleogelation process. Additionally, this group was the only group where there was a significant statistical difference in enthalpy. SP70MG and SP70HF showed a higher enthalpy, and SP70LE had a lower enthalpy compared to SP70 alone.

### 2.5. X-ray Diffraction

**Figure 4:**
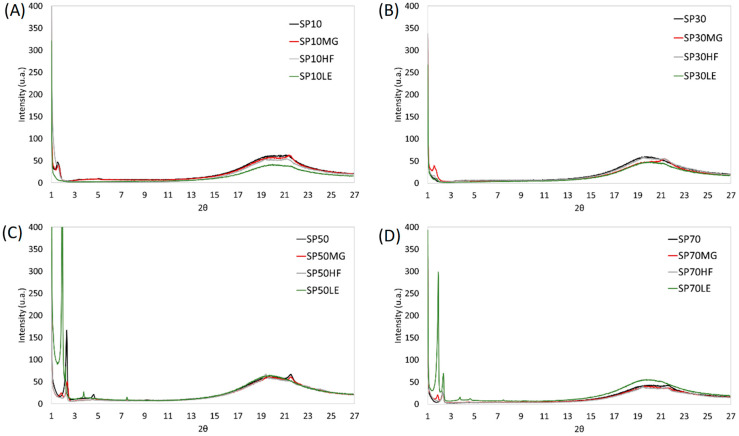
X-ray diffraction of the oleogels structured using sucrose esters (**A**) SP10, (**B**) SP30, (**C**) SP50 and (**D**) SP70 and their blends with monoglycerides (MGs), hard fat (HF) and lecithin (LE).

Figure 4 presents the X-ray profiles of the SE oleogels. Similar to previous observations, SE, regardless of the SE used, exhibited only one diffraction peak at 4.15 Å in the wide-angle region, and this was true for all samples [14]. As published in our previously manuscript [38], the X-ray of the wide-angle MG-oleogel showed peaks at 4.6, 4.35, and 3.9 Å; HF-oleogel showed diffraction patterns at 4.6, 4.2, and 3.8 Å; LE-oleogel showed no pattern.

The investigated blends have different profiles depending on the components. HF blends showed peaks at 4.6 Å and 4.2 Å, except for SP50HF, which only presented a 4.6 Å diffraction peak. These were major peaks in the HF, which suggests that the oleogels of HF and SE are tailored by the HF structuration. MG blends, on the other hand, only presented a peak at 4.15 Å, except for SP30MG, where the peak was 4.2 Å, as was true for the SP30HF. This might be due to the absence of patterns found for SP30 alone. Regarding LE blends, although very small diffraction patterns started to appear at 4.15 Å for blends with SP10 and SP30, the intensity of the peak was extremely low and not present in blends with SP50 and SP70. These results suggest the predominance of SE crystalline patterns in these blends. SE shows a similar packing to the α and β’ phases of triacylglycerols [54]. Thus, SE, when blended with HF, corresponds to β + β’ form and other blends and oleogels based on SE alone in α-form.

Samples SP10 and SP30 in the small-angle region showed only one pattern at a distance of 53.4 Å (001 peaks). SE molecules were reported to arrange in bilayers of thickness d ≈ 50 Å, as shown by the small-angle scattering lines [47]. Conversely, higher HLB samples (SP50 and SP70) displayed an important peak at 36 Å (002), meaning that the blends maintained the main pattern of the SE, either 53.4 Å (SP10 and SP30) or 36 Å (SP50 and SP70). Additionally, LE and MG added a peak at 47.5 Å on SP50 and SP70.

## 3. Conclusions

SEs with different HLBs can form synergic binary combinations when using alternative routes to structure the oleogel. Well-entrapped gels, with good physical properties, can be found using the SP70 (HLB 15) and MG/HF after using the foam-template approach to form oleogels. The foam template approach favors the solubility of SP70 in oil and the connections with high-melting-point triacylglycerols and MG.

Although SP50 (HLB 11) obtained using the ethanol route, when combined with MG, showed good mechanical properties, the network that was formed is not very strong, resulting in a high rate of oil loss. Moreover, the direct dispersion of low HLB SE does not form satisfactory oleogels alone or in binary mixtures with MG, HF, and LE.

## 4. Materials and Methods

### 4.1. Materials

SE (SP10, SP30, SP50, and SP70) samples were provided by Sisterna (Roosendaal, the Netherlands). SP10 (HLB 2, 10% monoester) is sucrose oligo ester with stearate/palmitate fatty acids. SP30 (HLB 6, 30% monoester) is a stearate/palmitate SE. SP50 (HLB 11, 50% monoester) is a stearate/palmitate SE, and SP70 (HLB 15, 70% monoester) is also a stearate/palmitate SE. Monoglycerides (MG, Dimodan^®^HP MB, ≥90% pure) were kindly donated by Royale Lacroix (Flémalle, Belgium). Lecithin (LE), Lipoid P 100 (Phosphatidylcholine 90% from soybean (non-GMO)), was obtained from Lipoid AG (Ludwigshafen, Switzerland). The fully hydrogenated rapeseed oil, hard fat (HF), was kindly donated by Cargill, Düsseldorf, Germany, and the rapeseed oil (RO) was acquired from Royale Lacroix (Flémalle, Belgium).

### 4.2. Oleogel Preparation

Oleoges were prepared using SEs alone or in binary combination with MG, HF, or LE. The total oleogelator concentration was always fixed at 10% (*w*/*w*) in RO. According to Dassanayake et al. [55], the total amount of oleogelators should not exceed 10% (*w*/*w*). The oleogel preparation was based on our previous research [14]. In this work, the oleogels from SEs (SP10, SP30, SP50, and SP70) were structured using three different routes: (1) TRADITIONAL (direct dispersion), (2) ETHANOL (solvent exchange), and (3) FOAM (foam template). It was found that each SE has an optimum structuration route. Based on these findings, the SP10 and SP30 (low HLB) were prepared using the traditional route or direct dispersion, the SP50 (medium–high HLB) was prepared using the ethanol solvent exchange route, and the SP70 (high HLB) was prepared using the foam template, as described below.

Samples SP10 and SP30 and their blends: oleogels were prepared by melting the SE alone or with MG, HF, or LE in pre-warmed RO (90 °C) using magnetic agitation (350 rpm) for 40 min, until complete dissolution of the oleogelator. Subsequently, the oleogel samples were statically cooled to 20 ± 0.05 °C at about 0.5 °C/min, and further stored for 48 h at 20 °C in an incubator.

SP50 and their blends: the SE was first diluted in 20 mL of ethanol 97%. The blend of ethanol + SE was additionally dispersed in pre-heated RO (45 mL, at 90 °C). Samples were then kept at 90 °C for one hour to allow ethanol evaporation. After ethanol evaporation, MG, HF, or LE was added and stirred for 40 min at 350 rpm using a magnetic. After blend dissolution samples were cooled to 20 °C and statically to 20 ± 0.05 °C at approximately 0.5 °C/min, and then stored for 48 h at 20 °C.

Sample SP70 and blends were produced using the foam-template approach based on Patel et al. [6]. SP70 was initially dispersed in 500 mL water (2 *w*/*w*), using magnetic agitation (500 rpm) for 5 h at 20 ± 2 °C. Afterwards, the mixture water + SP70 was agitated with an ultra-turrax (IKA, Werke, Germany) at 11,000 rpm for 5 min and then frozen in an aluminum dish overnight at −50 °C. The frozen aqueous blend was freeze-dried for 72 h. The obtained powder was used to produce oleogels by direct dispersion at room temperature (20 ± 0.05 °C, SP70 alone). To produce the blend, samples were first structured by MG, HF, or LE using direct dispersion (90 °C, 20 min, 350 rpm stirring). During the cooling step (0.05 °C/min), before visual crystallization of the oleogelators, the SP70 foam powder was added (45 °C, for MG and HF, and 20 °C for LE); blends were mixed using a magnetic stirrer (100 rpm) and stored in a 20 °C incubator for 48 h before further analysis. All oleogels were produced in triplicate.

### 4.3. Methods

#### 4.3.1. Microscopy

Polarized light microscopy (PLM) analysis was performed using an optical microscope (Nikon Eclipse E400, Kanagawa, Japan) equipped with a digital camera (Nikon, DS-Fi2, Tokyo, Japan). The slides were prepared by adding one drop of the stabilized sample and covering with a cover slide. One slide of each oleogel replicate was performed, and four images of each slide were taken from different spots.

#### 4.3.2. Oil Loss

Oil loss was measured using a 5810R Eppendorf centrifuge (Hamburg, Germany). Approximately one gram of stabilized oleogel was filled on a pre-weighted 2 mL Eppendorf (*wa*). The Eppendorf with the sample was weighted before centrifugation (*wb*). After centrifugation, performed at 20 °C for 15 min at 2950 g, the sample was weighted only after draining the free oil by leaving it on its head for 10 min (*wc*). Equation (1) summarizes the oil loss calculation. Each sample was tested in quadruplicate.

Oil loss (%) = [(*wb* − *wc*)/(*wb* − *wa*) × 100] (1)

#### 4.3.3. Rheology

A modular compact rheometer MCR 302 (Rheoplus, Anton Paar, Graz, Austria) with a plate–plate 40 mm geometry and 1000 μm gap was used to measure strain sweeps (0.0008 to 100% strain at 1 Hz). The results of viscoelasticity (G′ and G″) were calculated in the linear viscoelastic region (LVR) using the Rheoplus software (Anton Paar, Graz, Austria). All samples were analyzed in quadruplicate at 20 °C.

#### 4.3.4. Texture

The sample hardness was evaluated by penetration test with a 5 mm diameter cylinder probe moving at 1 mm/s for 10 mm and using a texture analyzer (TAXT plus, Stable Micro Systems, Surrey, UK) equipped with a 5 kg load cell. The hardness reported here (N) corresponded to the force of the maximum peak. Analyses were carried out in triplicate.

#### 4.3.5. Melting Properties

A differential scanning calorimetry (DSC), Q2000 DSC (TA Instruments, New Castle, DE, USA) connected to a refrigeration cooling system (TA Instruments, New Castle, DE, USA) was used to measure the melting curves of the oleogels. Calibration of the DSC was carried out with indium and eicosane. A T-zero hermetic pan was used, where about 5–8 mg of oleogel was weighed and sealed. A similar T-zero hermetic empty pan was used as a reference. The melting curve was recorded while the heating the sample from 20 °C to 100 °C, at 5 °C/min. Analyses were performed in triplicate. Melting parameters, peak temperature (T_p_), and enthalpy (∆H) were calculated using the Universal Analysis Software version 4.2 (TA Instruments, New Castle, DE, USA).

#### 4.3.6. X-ray Diffraction (XRD)

A Bruker D8 Advance Diffractometer (Bruker, Karlsruhe, Germany) was used to measure X-ray diffraction. Measurements were performed using a LynxEye detector (Bruker, Germany), with a Cu Kα radiation (λ = 1.54178 Å, 40 kV, 30 mA) from 1 to 27° 2θ, with a 0.02° as the step size. The data analysis was carried out using the Diffrac.Eva software (Bruker, Karlsruhe, Germany).

#### 4.3.7. Statistical Analysis

Statistical analysis was calculated using 2-way ANOVA (α < 0.05), and correlation analyses were performed using GraphPad Prism version 8.0 (La Jolla, CA, USA). All data shown are mean value with a standard deviation of the mean.

## Figures and Tables

**Table 1:** Melting parameters of oleogels formed by sucrose esters (SP10, SP30, SP50 and SP70), and binary combinations of SE with monoglycerides (MG), hard fat (HF), and lecithin (LE).

Oleogelators	** T_p_ (°C)				ΔH (J/g)			
	-	MG	HF	LE	SP	MG	HF	LE
SP10	65.4 ± 0.1 ^a,^*	61.3 ± 0.2 ^b^	61.0 ± 0.3 ^b^	41.7 ± 1.1 ^f^	7.5 ± 0.7 ^a,b,c,d^	9.4 ± 1.0 ^a^	11.6 ± 0.7 ^a^	2.2 ± 0.2 ^e,f^
SP30	49.5 ± 0.2 ^d,e^	60.5 ± 1.2 ^b^	54.6 ± 0.2 ^c^	42.8 ± 0.9 ^f^	3.8 ± 0.3 ^d,e,f^	8.2 ± 0.4 ^a,b,c^	10.9 ± 0.9 ^a^	3.1 ± 0.5 ^e,f^
SP50	46.3 ± 1.0 ^e,^	37.2 ± 0.5 ^g^	49.3 ± 1.1 ^d,e^	34.1 ± 0.3 ^g^	4.7 ± 0.4 ^b,c,d,e,f^	4.2 ± 0.8 ^c,d,e,f^	7.6 ± 0.1 ^a,b,c,d^	5.4 ± 2.5 ^b,c,d,e^
SP70	51.0 ± 0.1 ^d^	51.8 ± 0.1 ^c,d^	51.6 ± 0.2 ^c,d^	51.7 ± 0.1 ^c,d^	5.4 ± 0.1 ^b,c,d,e^	10.8 ± 0.2 ^a^	8.7 ± 0.3 ^a,b^	0.9 ± 0.1 ^f^

* Samples followed by the same letter in the same column are not statistically different from each other based on Tukey test (α = 0.05). ** T_p_: peak temperature, ΔH: enthalpy.

## Data Availability

Not applicable.

## Supplementary Materials

The following supporting information can be downloaded at: https://www.mdpi.com/article/10.3390/gels9050399/s1, Figure S1: Macro and microstructure of the SEs (SP10, SP30, SP50 and SP70) and their blends with hard-fat (HF), lecithin (LE) and monoglycerides (MG), 40× magnification.
