# Supplementary material for: Sucrose Esters as Oleogelators in Mono or Binary Structured Oleogels Using Different Oleogelation Routes
Source: Gels. 2023 May 10;9(5):399. doi: 10.3390/gels9050399 (PMC10217855; doi:10.3390/gels9050399)
Supplement: Supplementary file 1 [file gels-09-00399-s001.zip › gels-2372176-supplementary.pdf]

Supplementary Material

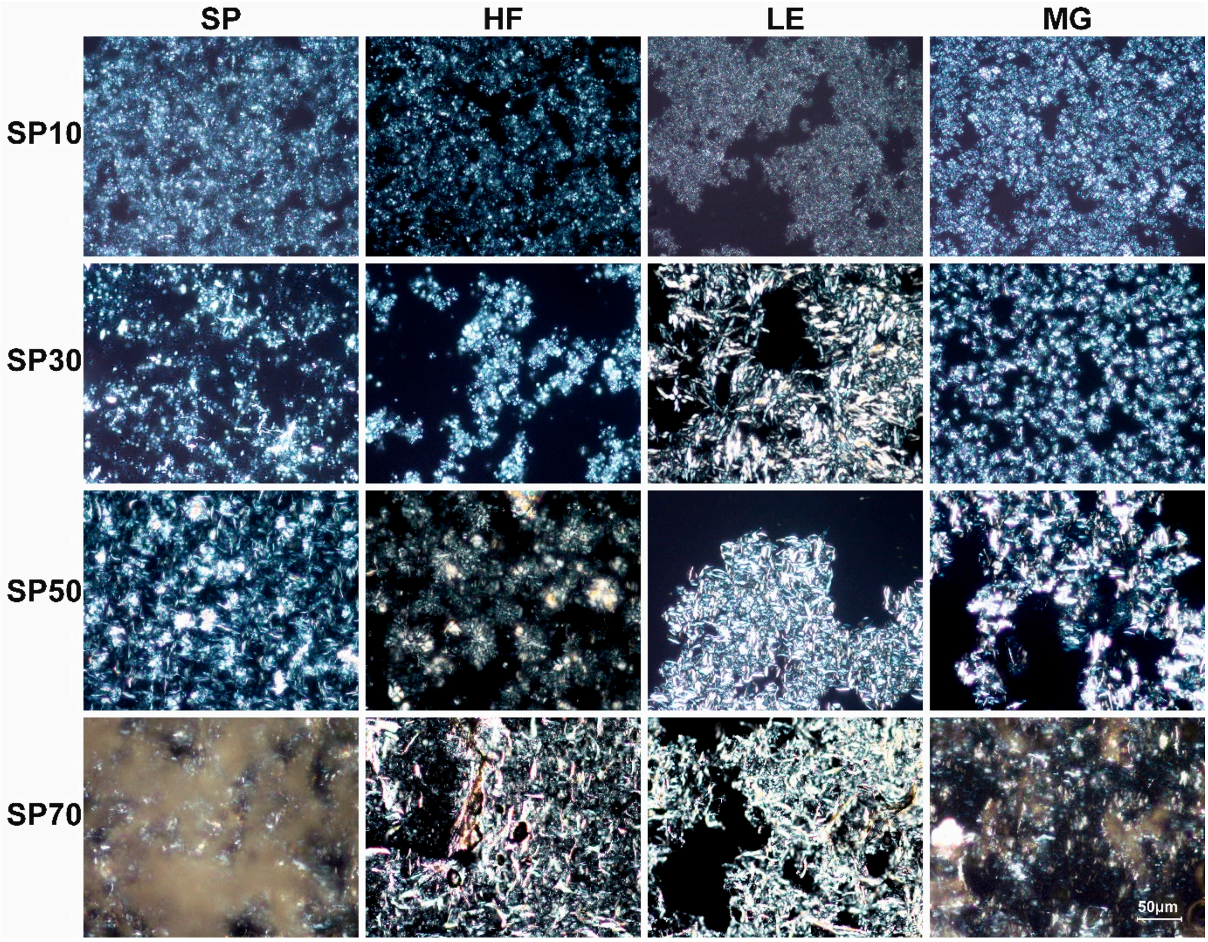

Figure S1. Macro and microstructure of the SEs (SP10, SP30, SP50 and SP70) and their blends with hard-fat (HF), lecithin (LE) and monoglycerides (MG), 40x magnification.
